# Endothelial dysfunction: a unifying hypothesis for the burden of cardiovascular diseases in sub-Saharan Africa

**DOI:** 10.5830/CVJA-2015-043

**Published:** 2015

**Authors:** Uchechukwu KA Sampson, Michael M Engelgau, Emmanuel K Peprah, George A Mensah

**Affiliations:** Center for Translation Research and Implementation Science, National Heart, Lung, and Blood Institute, National Institutes of Health, Bethesda, MD, USA; Center for Translation Research and Implementation Science, National Heart, Lung, and Blood Institute, National Institutes of Health, Bethesda, MD, USA; Center for Translation Research and Implementation Science, National Heart, Lung, and Blood Institute, National Institutes of Health, Bethesda, MD, USA; Center for Translation Research and Implementation Science, National Heart, Lung, and Blood Institute, National Institutes of Health, Bethesda, MD, USA

**Keywords:** endothelium, risk factors, cardiovascular disease, public health, outcomes

## Abstract

It is well established that the leading causes of death and disability worldwide are cardiovascular diseases (CVD), chief among which is ischaemic heart disease. However, it is also recognised that ischaemic heart disease frequently coexists with other vascular conditions, such as cerebrovascular, renovascular and peripheral vascular disease, thus raising the notion of a common underlying pathobiology, albeit with differing manifestations, dictated by the implicated vascular bed.

The understanding that common metabolic and behavioural risk factors as well as social determinants and drivers are convergent in the development of CVD evokes the idea that the dysfunction of a common bio-molecular platform is central to the occurrence of these diseases. The state of endothelial activation, otherwise known as endothelial dysfunction, occurs when reactive oxygen signalling predominates due to an uncoupled state of endothelial nitric oxide synthase (eNOS). This can be a physiological response to stimulation of the innate immune system or a pathophysiological response triggered by cardiovascular disease risk factors.

The conventional wisdom is that the endothelium plays an important role in the initiation, progression and development of CVD and other non-communicable diseases. Consequently, the endothelium has remarkable relevance in clinical and public health practice as well as in health education, health promotion, and disease- and risk-factor prevention strategies. It also presents a plausible unifying hypothesis for the burden of CVD seen globally and in sub-Saharan Africa. Importantly, the heterogeneity in individual responses to metabolic, behavioural, and social drivers of CVD may stem from a complex interplay of these drivers with genomic, epigenetic and environmental factors that underpin eNOS uncoupling. Therefore, further biomedical research into the underlying genetic and other mechanisms of eNOS uncoupling may enlighten and shape strategies for addressing the burden of CVD in sub-Saharan Africa and other regions of the world.

## Abstract

The understanding that hypertension, dyslipidaemia and tobacco use are powerful risk factors for the genesis of cardiovascular disease (CVD) has led to heavy investments in basic, clinical and population science research targeted at the prevention, treatment and control of CVD risk factors. Cumulative evidence from these research investments have informed clinical practice guidelines for the management of CVD, and largely account for the observed 60 to 80% decline in mortality from stroke and coronary artery disease in most developed nations in the past 50 years.^1^ In the United States, we have witnessed a 68% decrease in age-adjusted death rates from heart disease (from 56 to 18 per 10 000 population) and a 79% decrease in stroke death rates (from 18 to four per 10 000 population) between 1958 and 2010.^2^

A more recent notion based on convergent lines of experimental and pathological evidence is that inflammation may be the unifying factor in the pathobiology of atherothrombosis and its complications, as well as most vascular diseases.^3^ Herein, the aggregate of clinical trial evidence led to the coronation of statins as the undisputed heavy-weight champions of pharmacological strategies for modulating inflammation, over and beyond cholesterol levels, for primary and secondary prevention of CVD events.^4^ The risk factor and inflammation hypotheses were pivotal in helping us understand the decline in CVD mortality rates in industrialised nations. However, despite the remarkable progress in reducing CVD mortality rates, recent evidence indicates that ischaemic heart disease and stroke remain leading causes of mortality in the United States and worldwide.^5^

Further significant reduction in CVD mortality rates may require new transformative paradigms that can have broad impact. In this context, there are clues that point to the endothelium as the expansive entity that links various vascular-related diseases. Most CVD is caused by atherosclerosis initiated by the loss of functional integrity of the endothelium, which can affect various vascular beds, resulting in disease conditions such as coronary heart disease, peripheral arterial disease and cerebrovascular disease.

Furthermore, the fact that common social determinants, and behavioral and metabolic risk factors attend these diseases and other major non-communicable diseases (NCDs) suggests the presence of a common broad unifying entity that has wide spatial distribution. In this light, we consider the endothelium as the unifying feature/element for the burden of CVD in sub-Saharan Africa (SSA), because its functional integrity, which comprises the genotypic, phenotypic and environmental context of its existence, can predispose individuals or populations to CVD. In this regard, we discuss the public health relevance of the endothelium and the challenges and opportunities regarding the quest for transformative paradigms for reducing CVD burden.^6^,^7^

## The endothelium

Robert F Furchgott, Louis J Ignarro and Ferid Murad catalysed the wave of research that improved our understanding of endothelial function, which led to the joint award of the 1998 Nobel Prize in Physiology or Medicine ‘for their discoveries concerning nitric oxide as a signaling molecule in the cardiovascular system’. We now recognise that the healthy endothelium is in a quiescent state where nitric oxide (NO) produced by the endothelial isoform of nitric oxide synthase (eNOS) in its membrane-bound configuration is released, to silence cellular processes, by targeting cysteine groups in regulator molecules such as NFκB and the mitochondria.^8^

On the other hand, endothelial dysfunction is an activated state where the regulatory proteins such as NFκB and phosphatases are targeted by reactive oxygen species (ROS) produced from oxidases and eNOS uncoupling. Endothelial activation can occur physiologically in response to immune system perturbation, as well as pathophysiologically secondary to cardiovascular risk factors. Persistent ROS signalling precipitates a loss of vascular integrity characterised by detachment of endothelial cells and dependence on circulating progenitor cells for repair due to limited capacity of contiguous endothelial cells.^8^

The relationship between risk-factor profile, endothelial dysfunction and circulating endothelial progenitor cells has been evaluated using flow-mediated dilatation (FMD) of the brachial artery. In their report, Hill and colleagues demonstrated that the presence of high levels of endothelial progenitor cells preserves endothelial function despite significant risk-factor burden.^9^ Similarly, the relationships between FMD and coronary disease risk factors in asymptomatic adults,^10^ diet and exercise in overweight teenagers,^11^ and glucose and other metabolic syndrome components have been reported.^12^

Beyond the association with cardiovascular risk factors, other measures of endothelial function have been associated with cardiovascular disease outcomes. Greater event-free survival has been associated with intracoronary acetylcholine-induced vasodilatation in coronary angiography patients,^13^ increased brachial artery reactivity indexed by FMD in vascular surgery patients,^14^ and increased baseline levels of endothelial progenitor cells in CAD patients.^15^ Furthermore, there is evidence to indicate early risk-factor exposure and endothelial dysfunction impact on the development of atherosclerosis and subsequent cardiovascular outcomes.^16^,^17^

The promotion of endothelial health and reversal of endothelial dysfunction have been associated with increased physical activity, consumption of diets rich in fruit and vegetables, and avoidance of tobacco use or exposure to tobacco smoke.^18^-^24^ Consequently, the endothelium has remarkable relevance in clinical and public health practise as well as in health education, health promotion and prevention strategies, and therefore has implications for the epidemiological transition unfolding in developing world regions such as sub-Saharan Africa. In addition, it suggests that additional research into endothelial function, activation and dysfunction could provide novel proximal targets for clinical, public health and public policy interventions, in an effort to achieve maximum impact on population health.

## Public health relevance

The structure of the endothelium constitutes a remarkable feature, given its complexity, vast spatial distribution, and heterogeneity in different vascular beds. Combined with its role in the control of vasomotor tone, inflammation, homeostasis, endocrine and paracrine regulation, and cell growth, trafficking and survival,^25^ the endothelium has remarkable implications for CVD and other NCDs such as cancer, diabetes and chronic lung disease. Therefore, it is not surprising that endothelial biomedicine is recognised as a transdisciplinary field.

Population research evidence indicates that social determinants and drivers such as globalisation, urbanisation, ageing, income, education and housing are all linked with stress levels associated with CVD and other diseases, and connected with behavioral risk factors – unhealthy diet, tobacco use, physical inactivity and harmful use of alcohol, which are associated with metabolic risk factors such as high blood pressure, obesity, diabetes and raised blood lipid levels that ultimately lead to the manifestations of various diseases.

In a recent study of cardiovascular risk and events in 17 low-, middle- and high-income countries, it was noted that compared to high-income country populations, the risk factors for CVD were lower in low-income country populations, but disease outcomes were substantially worse, which potentially suggests both poor delivery of effective clinical care and higher stress levels in low-income country populations.^26^

**Fig. 1.**
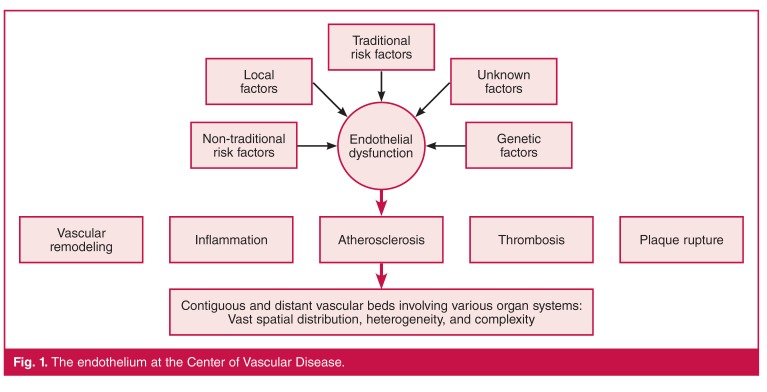
The endothelium at the Center of Vascular Disease.

The endothelium provides a construct for understanding how these networks of social, behavioural and metabolic factors converge to cause a network of diseases. The socio-behavioral and biological drivers lead to pathophysiological activation of the endothelium, resulting in a favourable bio-molecular milieu, for example inflammation and atherosclerosis, for disease in various vascular beds and organ systems due to the expansive spatial distribution of the endothelium (Fig. 1).

Therefore the endothelium provides a target for cross-cutting disease strategies given the broad implications of its dysfunction. Since moderate levels of physical activity on most days of the week, diets rich in fruit and vegetables and low in saturated and trans fats, and tobacco avoidance have been shown to improve endothelial health and reverse endothelial dysfunction, the adherence to public health strategies for improving physical activity and nutrition are essential for health promotion and the prevention of CVD, which aligns with clinical guideline-recommended interventions for the treatment and control of the common risk factors associated with CVD.

However, we need to move beyond current approaches by deliberately seeking transformative ways to achieve further substantial decline in CVD morbidity and mortality rates. Here, it is important to build on the wealth of scientific information on the endothelium, which has not been tapped by public health practitioners and researchers for translation into policies, programmes and research initiatives for advancing cardiovascular health promotion and the prevention of CVD.

## Challenges and opportunities

Although the endothelium establishes dialogue with every tissue cell in the body, is affected by many disease processes and risk factors, and contributes to the initiation and progression of chronic diseases, it remains underappreciated until it is dysfunctional. Furthermore, although measures to improve or preserve endothelial health are relatively inexpensive, they are often less supported than more expensive disease-intervention strategies.

This overall lack of prioritisation highlights the need for improved awareness, understanding and focus, particularly if we are to unlock the potential that exists for discovering endothelial targets that could significantly impact on CVD mortality rates. On this point, it is interesting that there is heterogeneity in response to metabolic, behavioural and social drivers for CVD. What if we could understand why some individuals succumb to CVD risk factors while others don’t? Why do we observe precocious CVD development in some subgroups exposed to the same drivers as the rest of a population? Why are there pockets of positive or negative deviance^27^,^28^ in CVD prevalence and outcomes within populations exposed to the same socio-ecological and bio-behavioural risk factors and drivers?

Unlocking the mysteries behind these puzzling differential responses may rest on targeted efforts to understand the complicated interplay between known disease drivers and the genomic and epigenetic mechanisms that underpin pathways of eNOS uncoupling. Such enlightenment could shape strategies for addressing the burden of CVD in sub-Saharan Africa and other regions of the world.

The above notion is paramount, given the prospect of finding a robust proximal target(s) that can transform our approach to CVD prevention and treatment, and therefore should prompt us to reconsider the current status quo regarding our scientific investments. Insight from the Emerging Risk factor Collaboration study indicates that we have to screen 400 to 500 people to prevent one CVD event over a period of 10 years.^29^ Such modest clinical benefits despite significant financial investments foster the increasing drum beat from camps that question the likelihood of meaningful clinical benefit from identifying and measuring biomarkers.

As a matter of fact, due to the vagaries of causality, some schools of thought now argue for fewer risk factors instead of more. Furthermore, although the search for independent risk factors in medical research has become necessary for the formulation of risk-stratification schemes, causality cannot be definitively ascertained, even in controlled clinical trials.^30^

In this context, we are beginning to learn that Mendelian randomisation studies can assess whether risk-factor associations are truly causal, or due to confounding or reverse causation. An illustrative NHLBI (National Heart, Lung, and Blood Institute) example of this is the large-scale Mendelian randomisation study of high-density lipoprotein (HDL) and the risk of myocardial infarction (MI), where the investigators found that low-density lipoprotein (LDL) is likely to be causally related to MI, whereas HDL is probably only a correlate.^31^ This may explain why LDL-lowering drugs (e.g. statins) reduce risk, whereas every large trial of HDL-increasing drugs has failed, including the NHLBI-funded AIM-HIGH trial.^32^

Tools such as Mendelian randomisation could help us make better strategic decisions about drug development, risk stratification and prediction when deployed via large-scale cohort studies to identify candidate undiscovered biological pathways (e.g. endothelial dysfunction) and insights into distinguishing causality versus correlation. However, the application of such tools to decipher robust genetic lynchpins of endothelial dysfunction will require interdisciplinary collaboration and demand for paradigms that can transform CVD prevention and treatment efforts.^33^ In this context, the H3Africa programme constitutes a platform to engender collaboration and employ synergy in intellectual enterprise to address novel research questions that will inform strategies for CVD diagnosis, treatment and prevention in sub-Saharan Africa.

## Conclusions

Over the past half century, CVD mortality has declined appreciably in developed countries, largely secondary to the risk-factor paradigm that implicated hypertension, cholesterol and smoking in the genesis of CVD. This risk-factor model led to targeted research, prevention and treatment efforts to combat these culprits. More recently, the modulation of inflammation has presented a unifying framework for achieving further substantive decline in CVD mortality rates.

In SSA, the age-adjusted mortality from CVD has not declined, and the regional burden of CVD is rising, albeit modestly, largely due to population growth, aging and the epidemiological transition. To address this challenge in SSA and the persistent impact of CVD and other NCDs in developed nations, we need revolutionary ideas or targets. It is in this regard that we recognise the endothelium, which plays a remarkable role in health and disease. Its relevance to CVD warrants increased awareness and appreciation in public health and practice. It is now understood that socio-ecological and bio-behavioural drivers converge to affect various types of CVD and NCD via endothelial dysfunction. Furthermore, the varied response to these risk-factor exposures suggests a more complicated relationship with the underlying mechanisms for endothelial dysfunction.

Targeted efforts to understand the genomic and epigenetic mechanisms underpinning eNOS uncoupling may help explain the differential response to disease drivers and perhaps provide robust targets for CVD prevention and treatment. This concept requires the type of resources and framework for collaboration offered by the H3Africa platform. In the interim however, widespread dissemination, adoption and implementation of proven interventions for the prevention and control of CVD risk factors that are also affordable and acceptable in the SSA context are strongly encouraged.

## References

[1] Capewell S, O’Flaherty M (2008). What explains declining coronary mortality? Lessons and warnings.. Heart.

[2] Marsh B Declining lethality.. The New York Times..

[3] Libby P, Ridker PM (2006). Inflammation and atherothrombosis from population biology and bench research to clinical practice.. J Am Coll Cardiol.

[4] Jahangir E, Fazio S, Sampson UK (2013). Incident diabetes and statins: the blemish of an undisputed heavy weight champion?. Br J Clin Pharmacol.

[5] (2014). Global, regional, and national age-sex specific all-cause and cause-specific mortality for 240 causes of death, 1990–2013: a systematic analysis for the Global Burden of Disease Study 2013.. Lancet.

[6] Mensah GA, Catravas JD, Engelgau MM, Hooper WC, Madeddu P, Ryan US (2007). Vascular endothelium summary statement VI: Research directions for the 21st century.. Vasc Pharmacol.

[7] Mensah GA (2007). Healthy endothelium: the scientific basis for cardiovascular health promotion and chronic disease prevention.. Vasc Pharmacol.

[8] Deanfield JE, Halcox JP, Rabelink TJ (2007). Endothelial function and dysfunction: testing and clinical relevance.. Circulation.

[9] Hill JM, Zalos G, Halcox JP, Schenke WH, Waclawiw MA, Quyyumi AA (2003). Circulating endothelial progenitor cells, vascular function, and cardiovascular risk.. N Engl J Med.

[10] Celermajer DS, Sorensen KE, Bull C, Robinson J, Deanfield JE (1994). Endothelium-dependent dilation in the systemic arteries of asymptomatic subjects relates to coronary risk factors and their interaction.. J Am Coll Cardiol.

[11] Woo KS, Chook P, Yu CW, Sung RY, Qiao M, Leung SS (2004). Effects of diet and exercise on obesity-related vascular dysfunction in children.. Circulation.

[12] Thomas GN, Chook P, Qiao M, Huang XS, Leong HC, Celermajer DS (2004). Deleterious impact of “high normal” glucose levels and other metabolic syndrome components on arterial endothelial function and intima-media thickness in apparently healthy Chinese subjects: the CATHAY study.. Arterioscler Thromb Vasc Biol.

[13] Halcox JP, Schenke WH, Zalos G, Mincemoyer R, Prasad A, Waclawiw MA (2002). Prognostic value of coronary vascular endothelial dysfunction.. Circulation.

[14] Gokce N, Keaney JF, Hunter LM, Watkins MT, Nedeljkovic ZS, Menzoian JO (2003). Predictive value of noninvasively determined endothelial dysfunction for long-term cardiovascular events in patients with peripheral vascular disease.. J Am Coll Cardiol.

[15] Werner N, Nickenig G (2006). Clinical and therapeutical implications of EPC biology in atherosclerosis.. J Cell Mol Med.

[16] Lloyd-Jones DM, Leip EP, Larson MG, D’Agostino RB, Beiser A, Wilson PW (2006). Prediction of lifetime risk for cardiovascular disease by risk factor burden at 50 years of age.. Circulation.

[17] Juonala M, Viikari JS, Laitinen T, Marniemi J, Helenius H, Ronnemaa T (2004). Interrelations between brachial endothelial function and carotid intima-media thickness in young adults: the cardiovascular risk in young Finns study.. Circulation.

[18] Brown AA, Hu FB (2001). Dietary modulation of endothelial function: implications for cardiovascular disease.. Am J Clin Nutr.

[19] Franco OH, Bonneux L, de Laet C, Peeters A, Steyerberg EW, Mackenbach JP (2004). The Polymeal: a more natural, safer, and probably tastier (than the Polypill) strategy to reduce cardiovascular disease by more than 75%.. Br Med J.

[20] Franzoni F, Ghiadoni L, Galetta F, Plantinga Y, Lubrano V, Huang Y (2005). Physical activity, plasma antioxidant capacity, and endothelium-dependent vasodilation in young and older men.. Am J Hypertens.

[21] Fuchsjager-Mayrl G, Pleiner J, Wiesinger GF, Sieder AE, Quittan M, Nuhr MJ (2002). Exercise training improves vascular endothelial function in patients with type 1 diabetes.. Diabetes Care.

[22] Hamdy O, Ledbury S, Mullooly C, Jarema C, Porter S, Ovalle K (2003). Lifestyle modification improves endothelial function in obese subjects with the insulin resistance syndrome.. Diabetes Care.

[23] Hennig B, Toborek M, McClain CJ (2001). High-energy diets, fatty acids and endothelial cell function: implications for atherosclerosis.. J Am Coll Nutr.

[24] Wildman RP, Schott LL, Brockwell S, Kuller LH, Sutton-Tyrrell K (2004). A dietary and exercise intervention slows menopause-associated progression of subclinical atherosclerosis as measured by intima–media thickness of the carotid arteries.. J Am Coll Cardiol.

[25] Hwa C, Sebastian A, Aird WC (2005). Endothelial biomedicine: its status as an interdisciplinary field, its progress as a basic science, and its translational bench-to-bedside gap.. Endothelium: J Endothelial Cell Res.

[26] Yusuf S, Rangarajan S, Teo K, Islam S, Li W, Liu L (2014). Cardiovascular risk and events in 17 low-, middle-, and high-income countries.. N Engl J Med.

[27] Krumholz HM, Curry LA, Bradley EH (2011). Survival after acute myocardial infarction (SAMI) study: the design and implementation of a positive deviance study.. Am Heart J.

[28] Bradley EH, Curry LA, Ramanadhan S, Rowe L, Nembhard IM, Krumholz HM (2009). Research in action: using positive deviance to improve quality of health care.. Implement Sci.

[29] Kaptoge S, Di Angelantonio E, Pennells L, Wood AM, White IR, Gao P (2012). C-reactive protein, fibrinogen, and cardiovascular disease prediction.. N Engl J Med.

[30] Brotman DJ, Walker E, Lauer MS, O’Brien RG (2005). In search of fewer independent risk factors.. Arch Intern Med.

[31] Voight BF, Peloso GM, Orho-Melander M, Frikke-Schmidt R, Barbalic M, Jensen MK (2012). Plasma HDL cholesterol and risk of myocardial infarction: a mendelian randomisation study.. Lancet.

[32] Boden WE, Probstfield JL, Anderson T, Chaitman BR, Desvignes-Nickens P, Koprowicz K (2011). Niacin in patients with low HDL cholesterol levels receiving intensive statin therapy.. N Engl J Med.

[33] Chatsuriyawong S, Gozal D, Kheirandish-Gozal L, Bhattacharjee R, Khalyfa AA, Wang Y (2013). Genetic variance in nitric oxide synthase and endothelin genes among children with and without endothelial dysfunction.. J Translational Med.

